# Inhibition of the type III secretion system by syringaldehyde protects mice from *Salmonella enterica* serovar Typhimurium

**DOI:** 10.1111/jcmm.14354

**Published:** 2019-05-08

**Authors:** Qianghua Lv, Xiao Chu, Xinyu Yao, Kelong Ma, Yong Zhang, Xuming Deng

**Affiliations:** ^1^ Department of Respiratory Medicine First Hospital, Jilin University Changchun China; ^2^ Key Laboratory of Zoonosis Research, Ministry of Education, College of Veterinary Medicine, Institute of Zoonosis Jilin University Changchun China

**Keywords:** anti‐infection, effector proteins, *Salmonella enterica* serovar Typhimurium, syringaldehyde, type Ⅲ secretion system

## Abstract

The invasiveness of *Salmonella enterica* serovar Typhimurium (*S*. Typhimurium) is closely associated with the *Salmonella* pathogenicity island (SPI)‐encoded type Ⅲ secretion system (T3SS), which can directly inject a series of effector proteins into eukaryotic cells to enable bacterial infection. In this study, syringaldehyde was identified as an effective inhibitor of the *S*. Typhimurium T3SS using an effector protein‐lactamase fusion reporter system. Syringaldehyde treatment could inhibit the expression of important effector proteins (SipA, SipB and SipC) at a concentration of 0.18 mM without affecting bacterial growth. Additionally, significant inhibition of bacterial invasion and cellular injury was observed following the syringaldehyde treatment in the co‐infection system of HeLa cells and *S*. Typhimurium. Furthermore, treatment with syringaldehyde provided systemic protection to mice infected with *S*. Typhimurium, reducing mortality (40.00%) and bacterial loads and relieving caecal damage and systemic inflammation. The results presented in this study indicate that syringaldehyde significantly affects T3SS activity and is a potential leading compound for treating *S*. Typhimurium infections.

## 1 INTRODUCTION

*Salmonella enterica* is Gram‐negative facultative intracellular bacterial pathogen that can cause a wide variety of diseases, ranging from intestinal inflammation to life‐threatening typhoid fever as a systemic infection.^1^, ^2^ When *Salmonella* is ingested through contaminated food and water, it is capable of causing a range of diseases, from gastroenteritis to potentially lethal systemic infections in a wide range of hosts, including humans.^3^ An estimated over a billion cases of *Salmonella* gastroenteritis occur annually, leading to approximately 3.5 million deaths worldwide.^4^ The worldwide harm to human health caused by *Salmonella* poses a serious challenge to the prevention and control of this disease.

*Salmonella enterica* serovar Typhimurium (*S.* Typhimurium) is arguably one of the most studied bacterial pathogens and a successful infection caused by this bacterium requires the delivery of effector proteins into host cells via two type III secretion systems (T3SSs) encoded by the distinct *Salmonella* Pathogenicity Islands 1 and 2 (SPI‐1 and SPI‐2).^5^, ^6^ Notably, the translocation of effector proteins via the SPI‐1‐associated T3SS is largely involved in facilitating bacterial invasion of host cells,^7^ whereas SPI‐2 T3SS‐delivered effector proteins are primarily associated with promoting intracellular survival and replication.^8^ In a search for bacterial effector proteins, a previous study observed that SipA was necessary for efficient bacterial entry into epithelial cells.^2^ The mutant* sipA* was also impaired in its ability to be internalized by cultured epithelial cells after short infection times.^9^ The transport of bacterial effector proteins across the host cell membrane is facilitated by a subset of *Salmonella* effector proteins known as translocators, including SipB, SipC and SipD.^10^, ^11^ These proteins form a channel in the host cell membrane that enables the passage of bacterial effector proteins into the host cell.^10^ Studies have shown that the complex formed by SipC and SipB is essential for the insertion of both proteins into the host cell membrane during *Salmonella* invasion.^12^ Several trials have shown that mutations in genes of the *inv* operon, such as *invA* and *invG*, result in a reduction of virulence and colonization of *Salmonella* by the oral route.^13^, ^14^ The complexity of this molecular machine makes fighting infections a challenge.

The high incidence of antibiotic resistance in this pathogen makes the use of traditional therapies inefficient.^15^ Innovative therapeutic strategies aimed at inhibiting the T3SS of *S.* Typhimurium as well as alternatives to classical antibiotics are highly attractive, as they may reduce the severity of infection and improve antibacterial immune responses as well as representing attractive therapies for antibiotic‐resistant bacteria with a similar virulence system. Thus, T3SS is increasingly being proposed and explored as an attractive drug target for developing novel antibacterial agents.^15^, ^16^ We recently used a protein translocation assay and Western blotting to screen a series of natural plant compounds for their effect on the translocation and expression of T3SS effector proteins in vitro and identified a novel small terpenoid inhibitor, syringaldehyde, that effectively suppressed the activity of the T3SS in S. Typhimurium. In addition, we determined that syringaldehyde reduced the mortality and bacterial loads and relieved caecal damage and systemic inflammation in vivo. The preliminary mechanism of action determined for syringaldehyde is also discussed. In summary, syringaldehyde shows strong potential as a leading compound to inhibit the T3SS and pathogenicity of *S*. Typhimurium.

## 2 MATERIALS AND METHODS

### 2.1 Bacterial strains and reagents

The *S.* Typhimurium strain SL1344 and the *invA* mutant used in this study were kindly provided by Profs. Zhaoqing Luo and Daoguo Zhou from the Immunology and Infectious Diseases laboratory and the Department of Biological Sciences at Purdue University respectively. In addition, we used a *S.* Typhimurium strain expressing a SipA‐lactamase fusion and *S*. Typhimurium SipA‐3×Flag and SipB‐3×Flag strains that were constructed by our laboratory.

Unless otherwise specified, the *S.* Typhimurium strains were cultured at 37°C in Luria‐Bertani (LB) medium containing 0.3 m NaCl. All of the candidate drugs (purity > 98%) were purchased from Chengdu Heibpurify Co., Ltd. (Chengdu, China) and were dissolved in dimethyl sulphoxide (DMSO, Sigma‐Aldrich, United States).

### 2.2 Drug screening and protein translocation assay

HeLa cells were maintained in Dulbecco's Modified Eagle Medium (DMEM, Hyclone，United States) supplemented with 10% foetal bovine serum (FBS, Biological Industries, Israel) at 37°C in a 5% CO_2_ incubator. HeLa cells were plated in 96‐well plates at a density of 1.5×10^4^cells/well and were incubated for 16‐18 hours before infection.

Overnight cultures of *S*. Typhimurium expressing the SipA‐lactamase fusion were diluted 1:20 into fresh LB with different concentrations of candidate drugs in the culture medium. After shaking for 4 hours, the SL1344 or *invA* mutant strains were used to infect the HeLa cells at a multiplicity of infection (MOI) of 50 and were incubated for 1 hour. After washing with phosphate‐buffered saline (PBS) three times, the cells were covered with PBS containing 20 μl of 6 × CCF4/AM (Life Technologies, United States). The reaction was allowed to proceed for 90 minutes at room temperature prior to visual detection with an Olympus IX‐81 fluorescence microscope.

### 2.3 Effector protein expression assay

The expression of effector proteins was assayed by Western blotting. The *S*. Typhimurium strains (SL1344, SipA‐3×flag and SipB‐3×flag) were grown overnight in LB broth. Next, the cells were subcultured (1:100) into fresh LB and were grown for 4 hours with different concentrations of syringaldehyde. Subsequently, cells from specific volumes of the cultures (determined based on OD600_nm_measurements) were collected by centrifugation at 12 000 rpm for 5 minutes. The cell pellets were resuspended in 1 × sodium dodecyl sulphate (SDS)‐loading buffer and boiled for 5 minutes, after which the samples were separated by 10% SDS‐PAGE and transferred onto a PVDF membrane. Immunoblotting analyses were performed using the appropriate primary antibodies (an anti‐Flag‐tag mouse monoclonal antibody (diluted 1:2000) for SipA‐3 × flag and SipB‐3 × flag and an anti‐SipC rabbit polyclonal antibody (The anti‐SipC polyclonal antibody prepared and preserved in our laboratory, diluted 1:1000) and secondary antibodies (a peroxidase‐conjugated Affinipure goat anti‐mouse IgG (H + L) (diluted 1:2000) for SipA‐3×flag and SipB‐3×flag and a peroxidase‐conjugated Affinipure goat anti‐rabbit IgG (H + L) (diluted 1:2000) for SipC). The metabolic enzyme isocitrate dehydrogenase (ICDH) was used as internal control according the previous study.^17^ The blots were detected using the enhanced chemiluminescence (ECL) method and were scanned with a Tanon‐4200 Image System (Tanon, China).

### 2.4 Growth curve assay

An overnight culture was diluted 1:20 into 100 ml of LB and cultured at 37°C with shaking until reaching an OD_600nm_ of 0.3, after which the culture was divided into five Erlenmeyer flasks with specific final concentrations of syringaldehyde (0 ~ 0.72 mmol/L). Subsequently, the cultures were grown at 37°C with shaking and the OD600_nm_ values were measured with a spectrophotometer every 30 minutes.

### 2.5 Lactate dehydrogenase release assay

HeLa cells were plated into 96‐well plate at a density of 2×10^4 ^cells/well and were incubated at 37°C in a 5% CO_2_ incubator for 16‐18 hours before infection. After washing the cells three times with PBS, they were infected at an MOI of 100 with *S*. Typhimurium and cultured with different concentrations of syringaldehyde for 5 hours in DMEM. Subsequently, the cultures were centrifuged at 1000 rpm for 10 min and the LDH release was then measured in culture supernatants on a microplate reader at 490 nm using an LDH Cytotoxicity Assay Kit according to the manufacturer's instructions (Roche, Switzerland). The percentage of LDH release was calculated as described in a previous study.^17^ The LDH release from the HeLa cells incubated with syringaldehyde was measured at an effective concentration range (0.0225‐0.18 mmol/L) in independent tests to evaluate the cytotoxicity of syringaldehyde.

### 2.6 Adhesion, invasion, replication assay and immunofluorescence

The adhesion, invasion and replication of *S*. Typhimurium in Hela cells were verified using a gentamicin protection assay and was optimized according to the method described before.^18^, ^19^ HeLa cells were plated into 24‐well plates at a density of 5×10^5 ^cells/well. Overnight bacterial cultures were diluted 20‐fold into fresh LB with or without syringaldehyde at 37°C for 4 hours. After washing, HeLa cells were infected at a MOI of 100 and incubated at 37°C for 30 minutes. The HeLa cells were washed three times by PBS to clear away unadhered bacteria. Subsequently, the cells were permeabilized with 0.2% saponin for 10 minutes,^20^, ^21^ and the adhesive bacteria were counted by plating on LB agar plates. Following washing, save the plate labelled with ‘invasion’ for further treatment. DMEM containing 100 μg/ml gentamicin was added to each well of the plates in order to kill the extracellular *Salmonella*. The plates were incubated at 37°C, 5% CO_2_ for an additional 1 hour. After washing three times, the cells were permeabilized with 0.2% saponin for 10 minutes and the invasive bacteria were counted by plating on LB agar plates. Following washing, save the plate labelled with ‘replication’ for further treatment. DMEM containing 10 μg/ml gentamicin was added to the ‘replication’ plate to maintain clearance of extracellular *Salmonella* in the medium. The plate was incubated at 37°C, 5% CO_2_ for an additional 22.5 hours. After washing three times, the cells were permeabilized with 0.2% saponin for 10 minutes and the invasive bacteria were counted by plating on LB agar plates.

For immunofluorescence microscopy, a protocol was used based on a previous study,^22^ HeLa cells were infected at an MOI of 100 with *S*. Typhimurium SL1344 treated by 0.18 mmol/L syringaldehyde or *invA* mutant according to the gentamicin protection assay. After treatment with gentamicin, cells were fixed onto cover slips using 4% paraformaldehyde for 10 minutes and were incubated with an anti‐*S*. Typhimurium rabbit antibody (diluted 1:2000) for 1 hour. The cells were washed three times and then were incubated with the secondary antibody (Alexa Fluor 488, goat anti‐rabbit conjugated‐IgG, 1:1000 dilution) for 30 minutes. Next, the cells were permeabilized with 0.3% Triton X‐100 for 10 minutes and incubated with anti‐S. Typhimurium rabbit antibody (diluted 1:2000) for 1 hour. The cells were washed three times and then were incubated with the secondary antibody (Alexa Fluor 594, goat anti‐rabbit conjugated‐IgG, 1:1000 dilution) for 30 minutes. Finally, the cell nuclei were stained with DAPI and the immunofluorescence analysis was performed with an Olympus fluorescence microscope.

### 2.7 Quantitative real‐time PCR

The total RNA from each bacterial sample was isolated using the Trizol method described in a previous study.^23^ Approximately 1 μg of RNA was reverse‐transcribed to cDNA using TRANS *EasyScript* ^®^ One‐Step gDNA Removal and cDNA Synthesis Super Mix (Transgene, China) according to the manufacturer's instructions. The PCR reactions were performed in 20‐μl volumes using Fast Start Universal SYBR Green Master (Roche, Switzerland) as recommended by the manufacturer. The normalized levels of gene expression were calculated with the 2^−ΔΔCT^ method using gyrB gene as the endogenous control. The primer pairs used in this study are shown in Table 1.

**Table 1:** Primers used in this study

Primer	Sequence (5′‐3′)	Product size (bp)
*sipA*‐Forward	CCGGCACCTTGAAATGCAAA	388
*sipA*‐Reverse	CGAATCCACACGCGAATGAC	
*sipB*‐Forward	CATTGTGGTGGTCGCAGTTG	360
*sipB*‐Reverse	CGGGCGAGCATAAAATCAGC	
*sipC*‐Forward	CAGCTTCGCAATCCGTTAGC	358
*sipC*‐Reverse	TCAGCCTGGTTCAACGTCAG	
*hilA*‐Forward	ATGCCGTTCTGGTCATCCTG	340
*hilA*‐Reverse	AAGGGCGCATACTGCGATAA	
*hilD‐*Forward	TAACGTGACGCTTGAAGAGG	123
*hilD‐*Reverse	GGTACCGCCATTTTGGTTTG	
*hilC‐*Forward	AGCGTATCAAGTCTGAAGCG	147
*hilC‐*Reverse	ATCATAGCCACACATCGTCG	
*rstA‐*Forward	GGTTCTCTCATTGCCGCTTA	141
*rstA‐*Reverse	GCCATCTTTACCTGGAAGCA	
*invF‐* Forward	ATGGGTTTTGCTGAGTCCTG	119
*invF‐* Reverse	ACAGCGCCAGTACCTTATTG	
*gyrB*‐Forward	TCATTTCCACTACGAAGGCG	111
*gyrB*‐Reverse	CCGATACCGTCTTTTTCGGT	

### 2.8 Animal experiment

The acid environment of the stomach can be considered one of the host's first lines of defence against ingested bacteria. Prior to invasion, colonization and persistence within their host, *Salmonella* has evolved adaptive networks to protect themselves against the stress conditions. Therefore, whether the syringaldehyde planned for oral administration in this study has an effect on the acid stress response of *S*. Typhimurium remains to be verified. Before starting the animal experiment, bacteria survival test in artificially simulated gastric fluid (SGF) and relative mRNA transcription level of the acid resistance gene were performed according to the previous study.^24^, ^25^ The primers of targeted genes are shown in Table S1.

BALB/c female mice (6**‐**8 weeks‐old, 16‐18 g) were obtained from Changsheng Biotechnology Co. Ltd. (Liaoning, China) and were housed with a 12 hours light‐dark cycle. Water containing streptomycin (5 g/L) was provided before infection. For survival assays, oral gavage was performed with 5×10^7^ CFUs of either SL1344 or the *invA* mutant strain in 100 μl of saline solution, while the control group was administered the same volume of saline solution without bacteria. The treatments were performed administering 100 mg of syringaldehyde per kilogram of body weight three times one day prior to infection and at 8 hours intervals for another 5 days post‐infection.

To assess the bacterial loads in the spleens and livers, the specimens were homogenized in sterile phosphate‐buffered saline (PBS) and 10‐fold serial dilutions of the organ homogenates were plated onto streptomycin agar plates that were incubated for 12 hours at 37°C. Segments of the caeca were fixed in a 4% formaldehyde solution and embedded in paraffin for histological analysis. To evaluate the levels of cytokines (IL‐1β, IL‐6, TNF‐α and IFN‐γ) in the caeca, homogenized specimens were centrifuged for 10 minutes at 12 000 rpm at 4°C. The cytokine levels in the collected supernatants were measured using a commercial enzyme‐linked immunosorbent assay kit according to the manufacturer's instructions (Invitrogen). All animal experiments were performed in accordance with the animal use protocol issued by Jilin University (Protocol number: 20160315009).

### 2.9 Statistical analysis

The experimental data were assessed by unpaired two‐tailed *t* tests using GraphPad Prism 5.0 (GraphPad software, La Jolla, CA) and *P* values are indicated as follows: **P < 0.01, *P < 0.05.

## 3 RESULTS

### 3.1 Syringaldehyde inhibits the T3SS‐mediated translocation of a SipA‐lactamase fusion

**Figure 1:**
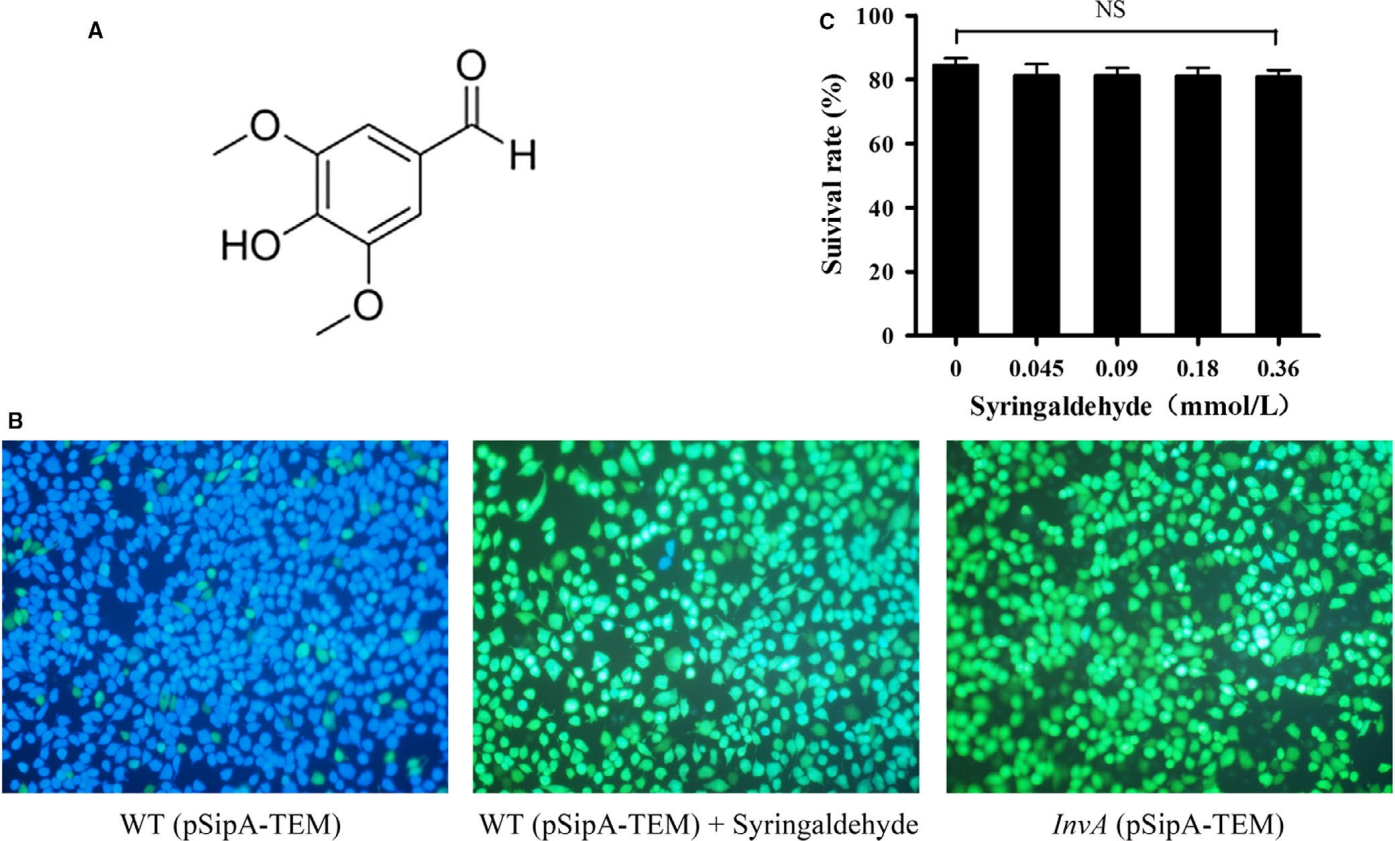
Syringaldehyde inhibits the translocation of effector proteins into host cells without affecting the bacterial growth. A, The chemical structure of syringaldehyde. B, Representative images of the translocation of the SipA‐lactamase fusion protein into HeLa cells, acquired with a fluorescence microscope. Wild‐type or *invA* mutant of *S.* Typhimurium SL1344 cells expressing the fusion protein was used to infect HeLa cells. Images showing blue and green cells indicate positive and negative protein translocation respectively. C, Toxicity of syringaldehyde to HeLa cells. Syringaldehyde was added to HeLa cells at the indicated concentrations and the LDH release was measured. The results shown are from three independent experiments performed in triplicate. Error bars represent standard errors of three datapoints. Significant differences between the syringaldehyde treatment and blank control groups are indicated (*P < 0.05, **P < 0.01)

To identify natural compounds capable of inhibiting translocation of the effector protein SipA into epithelial cells, a SipA‐TEM‐1‐β‐lactamase fusion reporter system was used as described previously.^2^ We showed that the terpenoid compound syringaldehyde (Figure 1A) was able to effectively inhibit the translocation of SipA into HeLa cells (Figure 1B). The release of lactate dehydrogenase was detected in the HeLa cells co‐incubated with the syringaldehyde after 5 hours. The results showed that the survival rate of host cells was not significantly different between the syringaldehyde treatment and blank control groups at a concentration of 0.36 mmol/L (Figure 1C), which exceeds the concentrations required for such inhibition. Taken together, the results demonstrated that syringaldehyde inhibited the translocation of the T3SS effector protein SipA without causing cytotoxicity towards HeLa cells in our assays.

### 3.2 Syringaldehyde inhibits T3SS effector protein expression through inhibition of SPI transcriptional regulation without affecting bacterial growth

**Figure 2:**
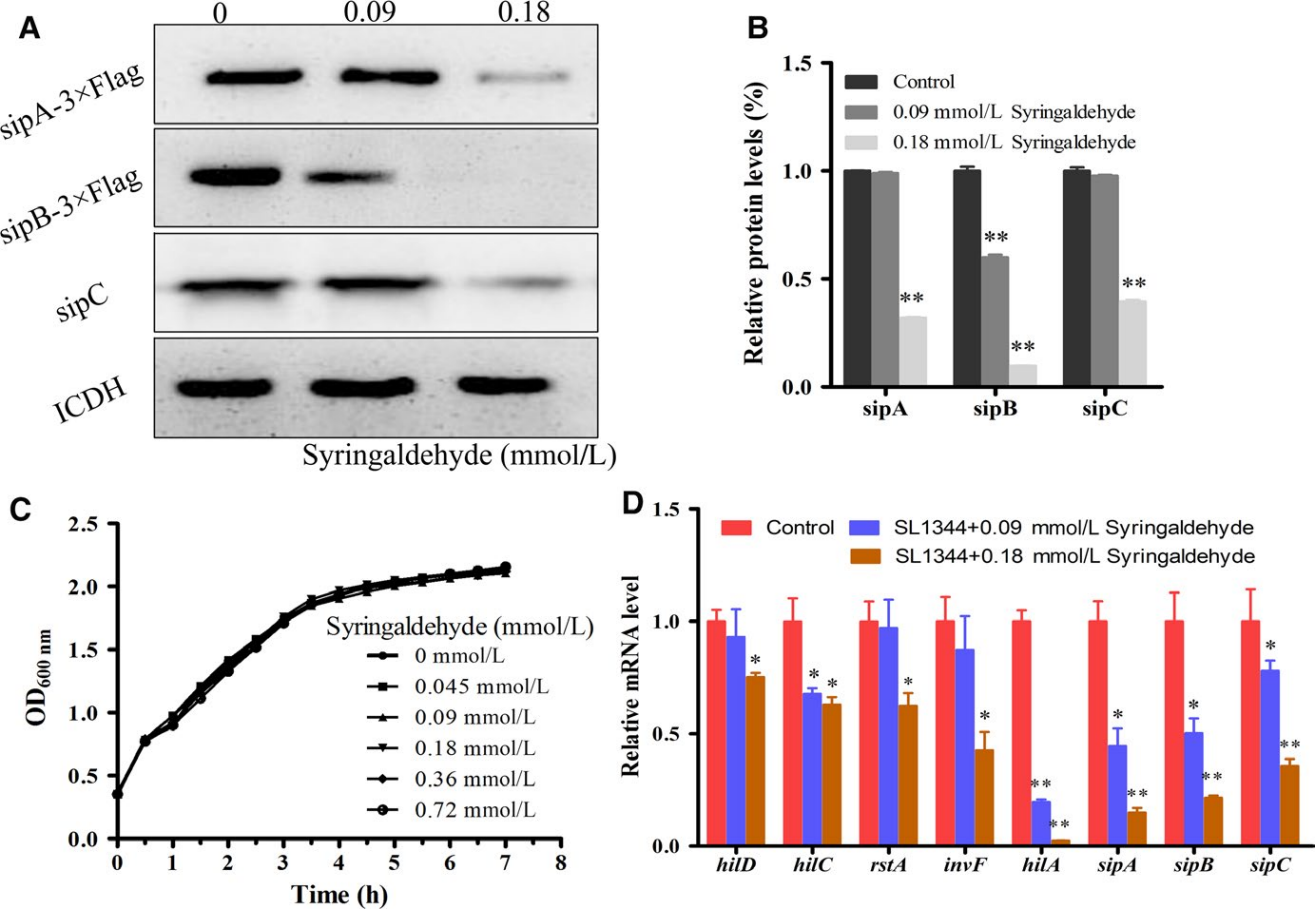
Syringaldehyde inhibits the expression of SPI‐1 T3SS effector proteins through inhibition of SPI transcriptional regulation. A, Strains expressing chromosomally encoded SipA‐3×Flag and SipB‐3×Flag were treated with the indicated concentrations of syringaldehyde for 4 h. The metabolic enzyme isocitrate dehydrogenase (ICDH) was used as internal control. Cultures of *S.* Typhimurium harbouring the SipA‐lactamase construct. After the co‐culture treatment with syringaldehyde and wild‐type SL1344, cell lysates were mixed with loading buffer, separated by SDS‐PAGE and detected with specific antibodies against SipC. Similar results were obtained from three independent experiments. B. Quantitative densitometry analysis of Western blotting using Image J analysis software. C. Growth curve of *S.* Typhimurium under co‐culture conditions with different concentrations of syringaldehyde. The bacterial growth status (OD_600nm_) was assessed every 30 min in LB medium containing the appropriate concentration of syringaldehyde until stationary phase was reached. The results shown are from three independent experiments performed in triplicate. D. Effect of syringaldehyde on the relative mRNA levels of the genes encoding SPI‐1 regulatory factors and effector proteins. RNA was extracted from bacteria treated with syringaldehyde at the appropriate concentration for 4 h. The y‐axis represents the relative transcriptional level of each gene (real‐time quantitative PCR) determined using the relative quantification method. Significant differences between the syringaldehyde treatment and blank control groups are indicated (*P < 0.05, **P < 0.01)

We assessed whether syringaldehyde has an effect on the expression of T3SS effector proteins (SipA, SipB and SipC) via a Western blotting assay. As shown in Figure 2A,B, syringaldehyde reduced the expression of the SipA‐3×Flag and SipB‐3×Flag fusion proteins and the effector protein SipC at a final concentration of 0.18 mmol/L. In addition, the growth of SL1344 was not significantly affected by syringaldehyde at a concentration of 0.72 mmol/L (Figure 2C). Thus, the syringaldehyde treatment inhibited the expression of effector proteins without affecting bacterial growth.

Given that the expression of various T3SS effector proteins was inhibited by syringaldehyde, we further assessed the expression of key regulatory and downstream effector genes at the transcriptional level via real‐time qPCR. Previous studies have shown that *hilD, hilC, rstA, invF, hil*A were key regulators of T3SS effector protein expression.^26^, ^27^, ^28^ Therefore, we investigated the expression of *hilD*, *hilC*, *rstA*, *invF*, *hilA*,* sipA*,* sipB* and* sipC* in the presence of syringaldehyde and the results showed that the transcription levels of these genes decreased when treated with syringaldehyde (Figure 2D). Taken together, these results showed that syringaldehyde inhibited the expression of effector proteins through SPI transcriptional regulation without affecting bacterial growth.

### 3.3 Syringaldehyde inhibits S Typhimurium‐mediated invasion of HeLa cells

**Figure 3:**
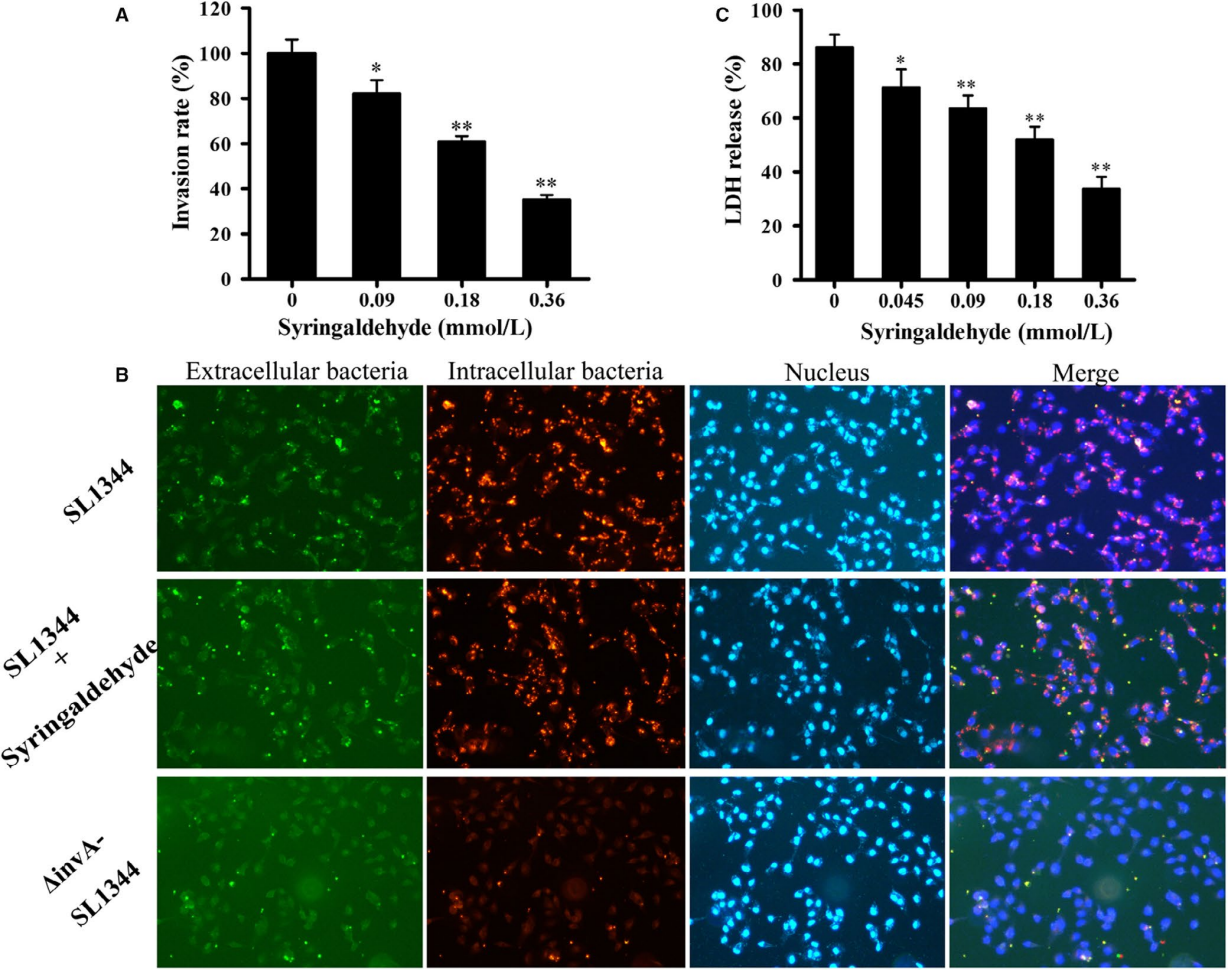
Syringaldehyde inhibits the invasion of HeLa cells by *S.* Typhimurium. A, The effects of syringaldehyde on the *S*. Typhimurium invasion of HeLa cells. Wild‐type *S*. Typhimurium SL1344 or the *invA* mutant was used to infect HeLa cells. Internalized bacteria were enumerated after killing extracellular bacteria with gentamicin. The rates of invasion were calculated by setting the values of internalized wild‐type bacteria in the blank control group samples as 100%. The results shown are from one representative experiment performed in triplicate. Significant differences between the syringaldehyde treatment and blank control groups are indicated (*P < 0.05, **P < 0.01). B, Representative images of bacterial invasion observed by immunofluorescence microscopy. Similar results were obtained with the previous experiments. Green indicates extracellular bacteria that were not internalized into the cell, red indicates intracellular bacteria that internalized into cells and blue indicates the host cell nucleus stained by DAPI. The merged images acquired for the relevant immunofluorescence signals indicate the colonization of bacteria in host cells. C, Syringaldehyde inhibits *Salmonella*‐mediated host cell damage. The results shown are from one representative experiment performed in triplicate by LDH release assay. Significant differences between the syringaldehyde treatment and blank control groups are indicated (*P < 0.05, **P < 0.01)

The above results indicated that syringaldehyde inhibits the expression of various *Salmonella* invasive proteins. Furthermore, we evaluated the inhibitory effects of syringaldehyde on the adhesion, invasion and replication of HeLa cells by *S*. Typhimurium SL1344 using a classic gentamicin protection assay. The results showed that syringaldehyde only inhibited the bacterial invasion of HeLa cells, meanwhile had no significant effect on bacterial adhesion and intracellular replication compared to the control group (Figure S1A,B). At a concentration of 0.18 mmol/L, the SL1344 invasion of HeLa cells was significantly inhibited, as the rate of bacterial internalization was reduced by 60.99 ± 0.05% (Figure 3A). *S*. Typhimurium invasion was also assessed through an immunofluorescence analysis, which yielded the same results (Figure 3B). These results demonstrated that the syringaldehyde treatment effectively blocked *S*. Typhimurium invasion of HeLa cells.

### 3.4 Syringaldehyde reduces S Typhimurium‐mediated HeLa cell injury

Previous studies have shown that *S.* Typhimurium SPI‐1 mutant strains are unable to damage eukaryotic cells.^29^ Therefore, we assessed the ability of syringaldehyde to protect HeLa cells from *S.* Typhimurium‐mediated damage. After co‐culturing SL1344 and HeLa cells with or without syringaldehyde the treatment for 5 hours, the LDH release in the culture supernatant was quantitatively evaluated using a microplate reader. Consistent with previous studies, most cells (86.08 ± 0.65%) died as a result of being infected with *S.* Typhimurium at a MOI of 100. However, the LDH release rate decreased to 33.67 ± 0.46% when the cultures were treated with 64 μg/ml of syringaldehyde (Figure 3C). Taken together, these results demonstrated that syringaldehyde can inhibit *S.* Typhimurium SL1344‐mediated HeLa cell injury.

### 3.5 Syringaldehyde increases the survival rate of mice infected with S Typhimurium

**Figure 4:**
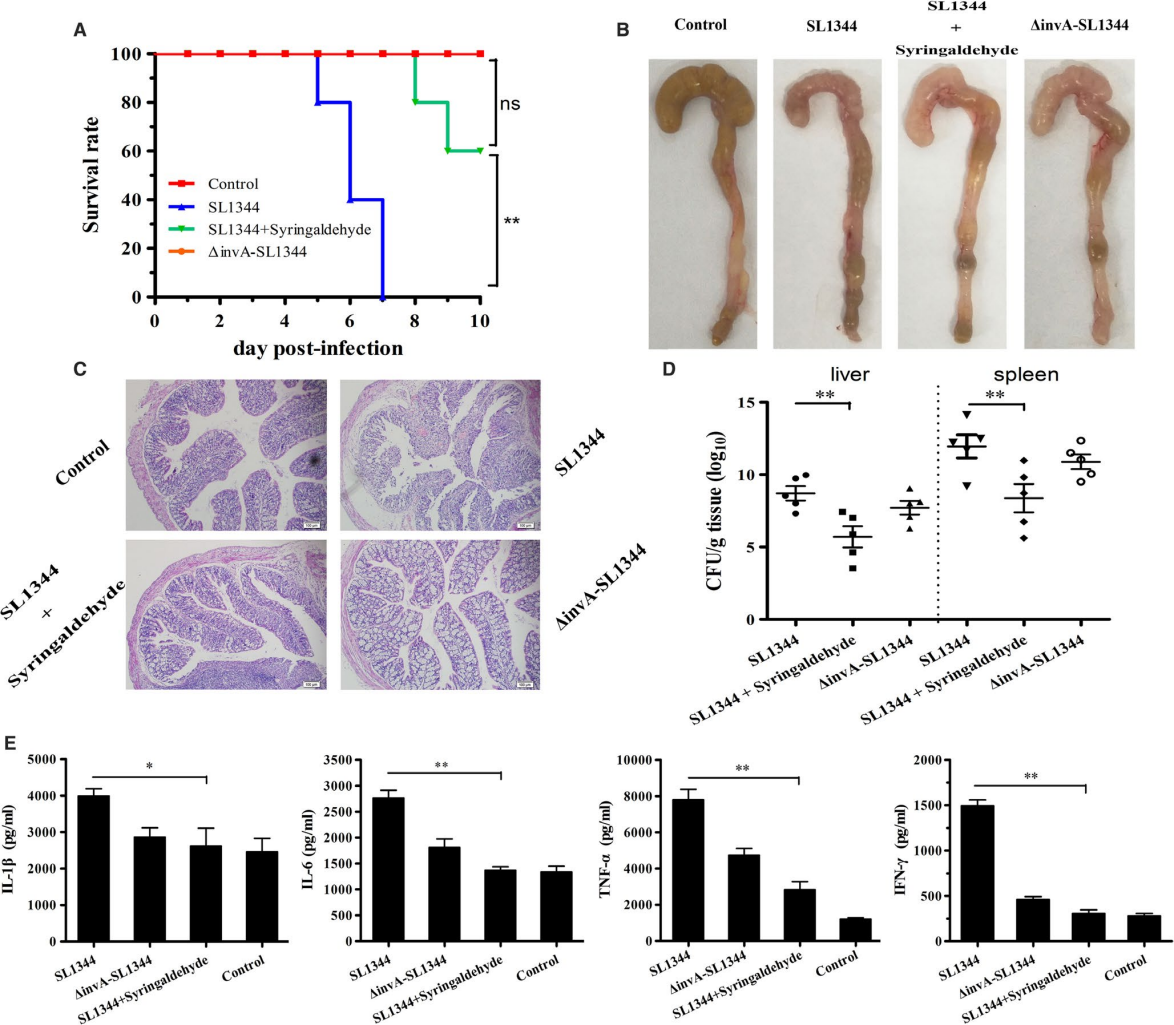
Protective effects of syringaldehyde on *S.* Typhimurium‐mediated mouse infection. A, Syringaldehyde prolongs the survival time and increases the survival rate of mice infected with *S.* Typhimurium. Wild‐type *S.* Typhimurium SL1344 and *invA* mutant strains used for oral gavage infection of BALB/c female mice. A dose of 5 × 10^7^ CFU bacteria of each strain was used to treat different groups of mice (n = 10), which were monitored for survival daily for 10 d after infection. B, Gross lesion observations of caecal tissues sections were prepared from each group. C, Histopathology observations of caecal tissue. The caecum tissues sections were prepared from each group and stained with haematoxylin and eosin. Finally, the caecal epithelial damage was evaluated by observations using an optical microscope. D, Syringaldehyde reduces the bacterial load in several organs of the infected mice. The bacterial load in the livers and spleens of mice was determined at 5 d after infection from tissue homogenate using the colony count method. The results shown are from three independent experiments performed in triplicate. E, In the syringaldehyde treatment group, cytokine production was reduced in the caecal tissue. All experiments were performed with caecal tissues from at least five mice and similar results were obtained from at least three independent experiments performed in triplicate. Error bars represent the standard errors of three datapoints. The level of significance is indicated as follows: *P < 0.05; **P < 0.01. The *P* value was calculated by comparing the values to that of the blank control group

In the *S.* Typhimurium strain SL1344 infection assay, no deaths were observed when mice were challenged with a high dose of the *invA* mutant (appropriately 5 × 10^7^CFUs) after 10 days. In contrast, 20.00% of the animals infected with the same dose of *S.* Typhimurium strain SL1344 died on the 5th day post‐infection and all mice succumbed to the infection at day 7. However, when mice were administered three 100 mg/kg doses of syringaldehyde at 8‐hour intervals the day prior to being challenged with *S.* Typhimurium strain SL1344, followed by identical doses at 8‐hour intervals for the next 5 days, no deaths were observed until the 7th day post‐infection. On the 8th day postinfection, 80.00% of the animals treated with syringaldehyde survived and 60.00% survived until day 10 post‐infection (Figure 4A). Thus, syringaldehyde prolonged the survival of *Salmonella*‐infected mice and significantly increased their survival rates.

To investigate the impact of the syringaldehyde treatment on the pathological manifestations of caecal injury, we performed histopathologic analyses of caeca from *S.* Typhimurium‐infected mice that received 100 mg/kg of syringaldehyde or 0.9% NaCl as a control. As shown in Figure 4B, caeca from the mice without the syringaldehyde treatment after being infected with wild‐type *S.* Typhimurium SL1344 exhibited significant damage, whereas tissue from mice treated with syringaldehyde displayed slight damage and inflammation that were similar to that observed in the control mice treated with 0.9% NaCl and those infected with the *invA* mutant. Notably, histopathological analyses showed that mice infected with wild‐type *S.* Typhimurium displayed submucosal oedemas, reduced numbers of goblet cells and intestinal villi that were largely shed and ruptured, whereas through histopathological analyses, mice treated with syringaldehyde or infected with *invA* mutant only displayed slight submucosal oedemas and reduced numbers of goblet cells that was similar to that observed in the control mice treated with 0.9% NaCl (Figure 4C). Similarly, syringaldehyde‐treated group significantly reduced the amount of bacteria associated with such organs as liver and spleen, indicating reduced bacterial dissemination in syringaldehyde‐treated mice (Figure 4D). As expected, the caecal histopathological results were supported by the cytokine levels observed in these tissue samples, which were significantly lower in the syringaldehyde‐treated group than in untreated mice (Figure 4E). The effects of syringaldehyde on the ability of S. Typhimurium to survive acid stress assay also showed that syringaldehyde had no significant effect on the acid resistance and the major acid resistance gene relative mRNA level of *S.* Typhimurium (Figure S2), which further proved that the protective effect of syringaldehyde on *Salmonella*‐infected mice was achieved by inhibiting T3SS. Taken together, our results showed that the syringaldehyde treatment provided systemic protection against *S.* Typhimurium a mouse infection model.

## 4 DISCUSSION

At present, the frequent occurrence of pathogens that are resistant to traditional antibiotics poses a great threat to public health, especially for developing countries with weak public health programs. Since the beginning of the 1990s, *Salmonella* strains exhibiting multidrug resistance, including resistance to fluoroquinolones, have emerged to the point where they now pose a serious public health problem,^30^ emphasizing the need for new control and treatment regimens. Therefore, antibiotic replacement therapies that target pathogenic virulence factors have become an innovative strategy. Major breakthroughs have been made in the study of many Gram‐negative bacteria, especially by targeting T3SS, which are key virulence factors in a large variety of Gram‐negative animal and plant pathogens.^15^ The newly developed drugs only block the function of certain virulence factors of pathogens to disarm the bacteria and this strategy has no effects on bacterial growth, potentially decreasing the likelihood of inducing drug resistance.

Increasing numbers of small molecule compounds have been identified in recent years with the aim of targeting specific key bacterial virulence factors,^31^ including those that affect the function of the bacterial type III secretion system. For instance, salicylidene acylhydrazides have a broad spectrum of inhibitory activity against T3SSs in several important Gram‐negative pathogens, including *Salmonella* spp, *Pseudomonas aeruginosa* and Chlamydiae.^32^, ^33^, ^34^ In addition, licoflavonol was shown to exhibit a strong inhibitory effect on the secretion and the transportation of SPI‐1 effector proteins.^35^

Although a large number of T3SS inhibitors have been reported, they have rarely been screened from natural plant extracts. In addition, the cost, limited source materials and unclear mechanisms of action of many synthetic drugs seriously restrict their development and practical application. SPI‐1 plays an important role during the early phase of infection and enable *salmonella* to penetrate the intact intestinal epithelial barrier and reach the subepithelial tissue.^36^ In this study, syringaldehyde was identified as a novel inhibitor against SPI‐1 without impact on *S*. Typhimurium growth and cytotoxicity at the mmol/L range in vitro. Additionally, syringaldehyde was analysed to have effects to suppress the transcription of the related key gene through the *hilD*‐*hilC*‐*rstA*‐*hilA* regulatory pathway to reduce the expression of the SPI‐1 genes encoding downstream effector proteins (SipA, SipB and SipC). Furthermore, syringaldehyde was shown to significantly prolong the survival time and improve survival rate (60.00%) of mice infected with *S*. Typhimurium and exhibiting anti‐inflammatory activity as determined by measuring levels of the cytokines IL‐1β, IL‐6, TNF‐α and IFN‐γ in a mouse model. Collectively, these results indicate that syringaldehyde provides comprehensive protection against *S*. Typhimurium infection.

Syringaldehyde is a traditional Chinese medicine obtained from a wide range of sources, having been identified and isolated from the stems of *Hibiscus taiwanensis* and others plants. As a terpenoid component of plant volatile oil, syringaldehyde (3,5‐dimethoxy‐4‐hydroxybenzaldehyde), has been shown to have hyperglycemic,^37^ anti‐oxidative and anti‐apoptotic activities.^38^ In addition, T3SS is conserved in many Gram‐negative pathogens, we showed that syringaldehyde is a potential inhibitor for developing natural small molecule compounds against bacterial pathogenesis in vitro and in vivo, further demonstrating that targeting the T3SS is a practical strategy. In summary, syringaldehyde may be an ideal leading compound with broad application to treat *Salmonella* infections in the future.

## CONFLICT OF INTEREST

The authors have no conflict of interest to declare.

## DATA AVAILABILITY STATEMENT

The data used to support the findings of this study are available from the corresponding author upon request.

## References

[1] Kurtz JR , Goggins JA , McLachlan JB . *Salmonella*infection: interplay between the bacteria and host immune system. Immunol Lett. 2017;190:42‐50. 28720334 10.1016/j.imlet.2017.07.006 PMC5918639

[2] Zhou D , Mooseker MS , Galan JE . Role of the *S. typhimurium* actin‐binding protein SipA in bacterial internalization. Science. 1999;283:2092‐2095. 10092234 10.1126/science.283.5410.2092

[3] Grenz JR , Cott Chubiz JE , Thaprawat P , Slauch JM . HilE regulates HilD by blocking DNA binding in *Salmonella enterica* serovar typhimurium. J Bacteriol. 2018;200. 10.1128/JB.00750-17 PMC5869468 29378886

[4] DeMasi L , Yue M , Hu C , Rakov AV , Rankin SC , Schifferli DM . Cooperation of adhesin alleles in salmonella‐host tropism. mSphere. 2017;2:e00066. 28289725 10.1128/mSphere.00066-17 PMC5343171

[5] Owen KA , Meyer CB , Bouton AH , Casanova JE . Activation of focal adhesion kinase by *Salmonella*suppresses autophagy via an Akt/mTOR signaling pathway and promotes bacterial survival in macrophages. PLoS Pathog. 2014;10:e1004159. 24901456 10.1371/journal.ppat.1004159 PMC4047085

[6] Jennewein J , Matuszak J , Walter S , et al. Low‐oxygen tensions found in Salmonella‐infected gut tissue boost *Salmonella*replication in macrophages by impairing antimicrobial activity and augmenting *Salmonella*virulence. Cell Microbiol. 2015;17:1833‐1847. 26104016 10.1111/cmi.12476

[7] Moest TP , Meresse S . *Salmonella*T3SSs: successful mission of the secret(ion) agents. Curr Opin Microbiol. 2013;16:38‐44. 23295139 10.1016/j.mib.2012.11.006

[8] Figueira R , Holden DW . Functions of the *Salmonella*pathogenicity island 2 (SPI‐2) type III secretion system effectors. Microbiology. 2012;158:1147‐1161. 22422755 10.1099/mic.0.058115-0

[9] Raffatellu M , Wilson RP , Chessa D , et al. SipA, SopA, SopB, SopD, and SopE2 contribute to *Salmonella enterica* serotype typhimurium invasion of epithelial cells. Infect Immun. 2005;73:146‐154. 15618149 10.1128/IAI.73.1.146-154.2005 PMC538951

[10] Myeni SK , Wang L , Zhou D . SipB‐SipC complex is essential for translocon formation. PLoS ONE. 2013;8:e60499. 23544147 10.1371/journal.pone.0060499 PMC3609803

[11] Glasgow AA , Wong HT , Tullman‐Ercek D . A Secretion‐amplification role for *Salmonella enterica* translocon protein SipD. ACS Synth Biol. 2017;6:1006‐1015. 28301138 10.1021/acssynbio.6b00335

[12] Kim BH , Kim HG , Kim JS , Jang JI , Park YK . Analysis of functional domains present in the N‐terminus of the SipB protein. Microbiology. 2007;153:2998‐3008. 17768243 10.1099/mic.0.2007/007872-0

[13] Murray RA , Lee CA . Invasion genes are not required for *Salmonella enterica* serovar typhimurium to breach the intestinal epithelium: evidence that *Salmonella*pathogenicity island 1 has alternative functions during infection. Infect Immun. 2000;68:5050‐5055. 10948124 10.1128/iai.68.9.5050-5055.2000 PMC101735

[14] Kaniga K , Bossio JC , Galan JE . The Salmonella typhimurium invasion genes invF and invG encode homologues of the AraC and PulD family of proteins. Mol Microbiol. 1994;13:555‐568. 7997169 10.1111/j.1365-2958.1994.tb00450.x

[15] Charro N , Mota LJ . Approaches targeting the type III secretion system to treat or prevent bacterial infections. Expert Opin Drug Discov. 2015;10:373‐387. 25727140 10.1517/17460441.2015.1019860

[16] Baron C . Antivirulence drugs to target bacterial secretion systems. Curr Opin Microbiol. 2010;13:100‐105. 20079679 10.1016/j.mib.2009.12.003

[17] Zhang Y , Liu Y , Wang T , Deng X , Chu X . Natural compound sanguinarine chloride targets the type III secretion system of *Salmonella enterica* Serovar Typhimurium. Biochem Biophys Rep. 2018;14:149‐154. 29761161 10.1016/j.bbrep.2018.04.011 PMC5948472

[18] Criss AK , Ahlgren DM , Jou TS , McCormick BA , Casanova JE . The GTPase Rac1 selectively regulates *Salmonella*invasion at the apical plasma membrane of polarized epithelial cells. J Cell Sci. 2001;114:1331‐1341. 11256999 10.1242/jcs.114.7.1331

[19] Wu J , Pugh R , Laughlin RC , et al. High‐throughput assay to phenotype *Salmonella enterica* Typhimurium association, invasion, and replication in macrophages. J Vis Exp. 2014;e51759. 25146526 10.3791/51759 PMC4500590

[20] Qiu J , Sheedlo MJ , Yu K , et al. Ubiquitination independent of E1 and E2 enzymes by bacterial effectors. Nature. 2016;533:120‐124. 27049943 10.1038/nature17657 PMC4905768

[21] Liu Y , Zhu W , Tan Y , Nakayasu ES , Staiger CJ , Luo ZQ . A legionella effector disrupts host cytoskeletal structure by cleaving actin. PLoS Pathog. 2017;13:e1006186. 28129393 10.1371/journal.ppat.1006186 PMC5298343

[22] Finn CE , Chong A , Cooper KG , Starr T , Steele‐Mortimer O . A second wave of *Salmonella*T3SS1 activity prolongs the lifespan of infected epithelial cells. PLoS Pathog. 2017;13:e1006354. 28426838 10.1371/journal.ppat.1006354 PMC5413073

[23] Zhou M , Guo Z , Yang Y , et al. Flagellin and F4 fimbriae have opposite effects on biofilm formation and quorum sensing in F4ac+ enterotoxigenic *Escherichia coli* . Vet Microbiol. 2014;168:148‐153. 24238669 10.1016/j.vetmic.2013.10.014

[24] Alvarez‐Ordonez A , Begley M , Prieto M , et al. *Salmonella*spp. survival strategies within the host gastrointestinal tract. Microbiology. 2011;157:3268‐3281. 22016569 10.1099/mic.0.050351-0

[25] Yang Y , Khoo WJ , Zheng Q , Chung HJ , Yuk HG . Growth temperature alters Salmonella Enteritidis heat/acid resistance, membrane lipid composition and stress/virulence related gene expression. Int J Food Microbiol. 2014;172:102‐109. 24368153 10.1016/j.ijfoodmicro.2013.12.006

[26] Brown NF , Rogers LD , Sanderson KL , Gouw JW , Hartland EL , Foster LJ . A horizontally acquired transcription factor coordinates *Salmonella*adaptations to host microenvironments. MBio. 2014;5:e01727‐e1814. 25249283 10.1128/mBio.01727-14 PMC4173766

[27] Eichelberg K , Galan JE . Differential regulation of *Salmonella*typhimurium type III secreted proteins by pathogenicity island 1 (SPI‐1)‐encoded transcriptional activators InvF and hilA. Infect Immun. 1999;67:4099‐4105. 10417179 10.1128/iai.67.8.4099-4105.1999 PMC96710

[28] Li J , Lv C , Sun W , et al. an inhibitor of the type III secretion system of *Salmonella enterica* serovar Typhimurium. Antimicrob Agents Chemother. 2013;57:2191‐2198. 23459474 10.1128/AAC.02421-12 PMC3632957

[29] Riquelme S , Varas M , Valenzuela C , et al. Relevant genes linked to virulence are required for *Salmonella*typhimurium to survive intracellularly in the social amoeba dictyostelium discoideum. Front Microbiol. 2016;7:1305. 27602025 10.3389/fmicb.2016.01305 PMC4993766

[30] Threlfall EJ . Antimicrobial drug resistance in Salmonella: problems and perspectives in food‐ and water‐borne infections. FEMS Microbiol Rev. 2002;26:141‐148. 12069879 10.1111/j.1574-6976.2002.tb00606.x

[31] Duncan MC , Linington RG , Auerbuch V . Chemical inhibitors of the type three secretion system: disarming bacterial pathogens. Antimicrob Agents Chemother. 2012;56:5433‐5441. 22850518 10.1128/AAC.00975-12 PMC3486574

[32] Negrea A , Bjur E , Ygberg SE , Elofsson M , Wolf‐Watz H , Rhen M . Salicylidene acylhydrazides that affect type III protein secretion in *Salmonella enterica* serovar typhimurium. Antimicrob Agents Chemother. 2007;51:2867‐2876. 17548496 10.1128/AAC.00223-07 PMC1932493

[33] Anantharajah A , Buyck JM , Sundin C , Tulkens PM , Mingeot‐Leclercq MP , Van Bambeke F . Salicylidene acylhydrazides and hydroxyquinolines act as inhibitors of type three secretion systems in pseudomonas aeruginosa by distinct mechanisms. Antimicrob Agents Chemother. 2017;61:e02566‐16. 28396545 10.1128/AAC.02566-16 PMC5444141

[34] Ur‐Rehman T , Slepenkin A , Chu H , et al. Pre‐clinical pharmacokinetics and anti‐chlamydial activity of salicylidene acylhydrazide inhibitors of bacterial type III secretion. J Antibiot. 2012;65:397‐404. 22669447 10.1038/ja.2012.43 PMC3428607

[35] Guo Z , Li X , Li J , et al. Licoflavonol is an inhibitor of the type three secretion system of *Salmonella enterica* serovar Typhimurium. Biochem Biophys Res Comm. 2016;477:998‐1004. 27387231 10.1016/j.bbrc.2016.07.018

[36] Laughlin RC , Knodler LA , Barhoumi R , et al. Spatial segregation of virulence gene expression during acute enteric infection with *Salmonella enterica* serovar Typhimurium. MBio. 2014;5:e00946‐e1013. 24496791 10.1128/mBio.00946-13 PMC3950517

[37] Kuo SC , Chung HH , Huang CH , Cheng JT . Decrease of hyperglycemia by syringaldehyde in diabetic rats. Horm Metab Res. 2014;46:8‐13. 23918689 10.1055/s-0033-1351274

[38] Bozkurt AA , Mustafa G , Tarik A , et al. Syringaldehyde exerts neuroprotective effect on cerebral ischemia injury in rats through anti‐oxidative and anti‐apoptotic properties. Neural Regen Res. 2014;9:1884‐1890. 25558237 10.4103/1673-5374.145353 PMC4281426

