# Supplementary figures and images for: Inhibition of the type III secretion system by syringaldehyde protects mice from Salmonella enterica serovar Typhimurium
Source: J Cell Mol Med. 2019 May 8;23(7):4679–88. doi: 10.1111/jcmm.14354 (PMC6584516; doi:10.1111/jcmm.14354)

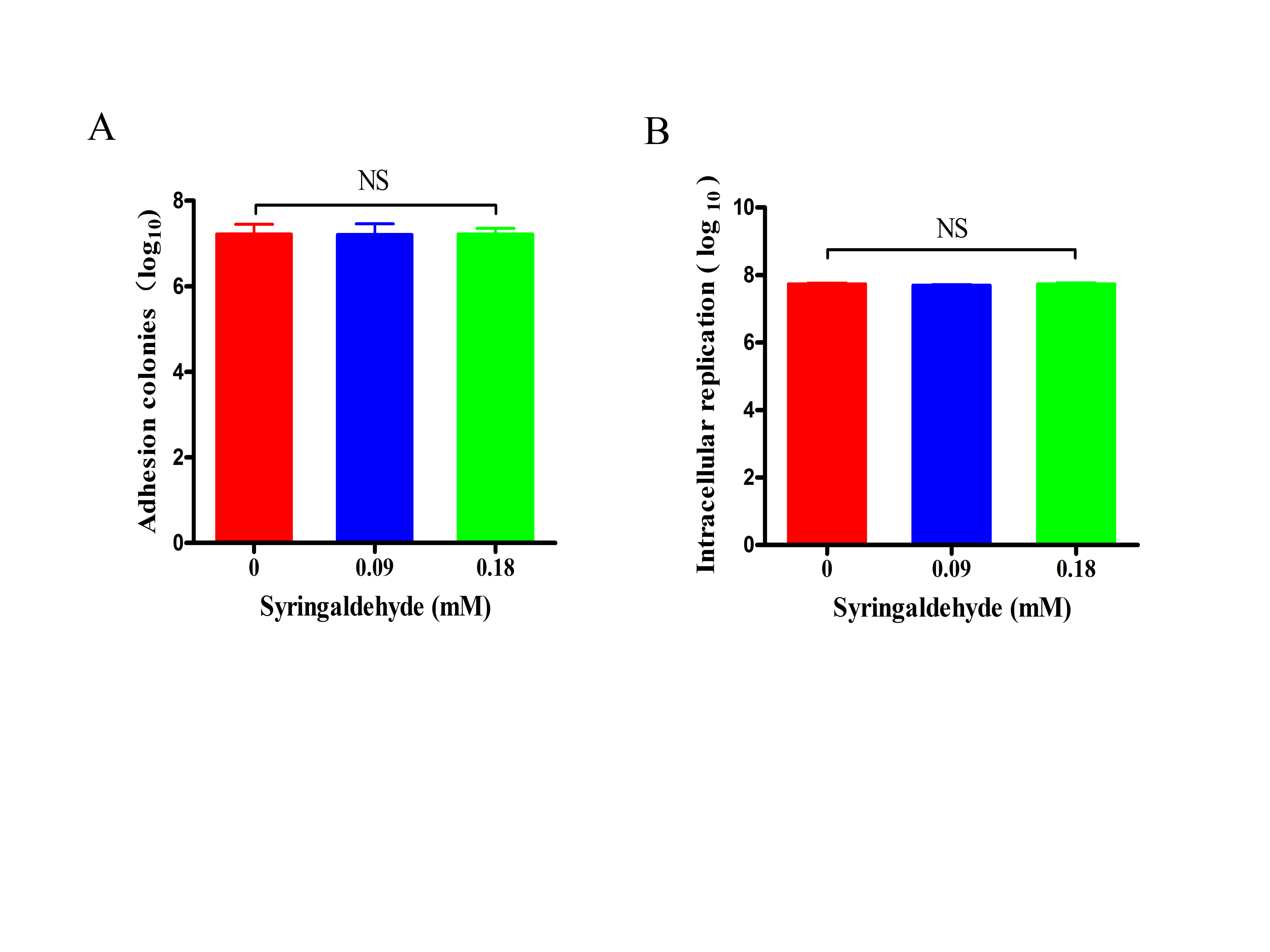

Supplement: Supplementary file 1 [file JCMM-23-4679-s001.tif]

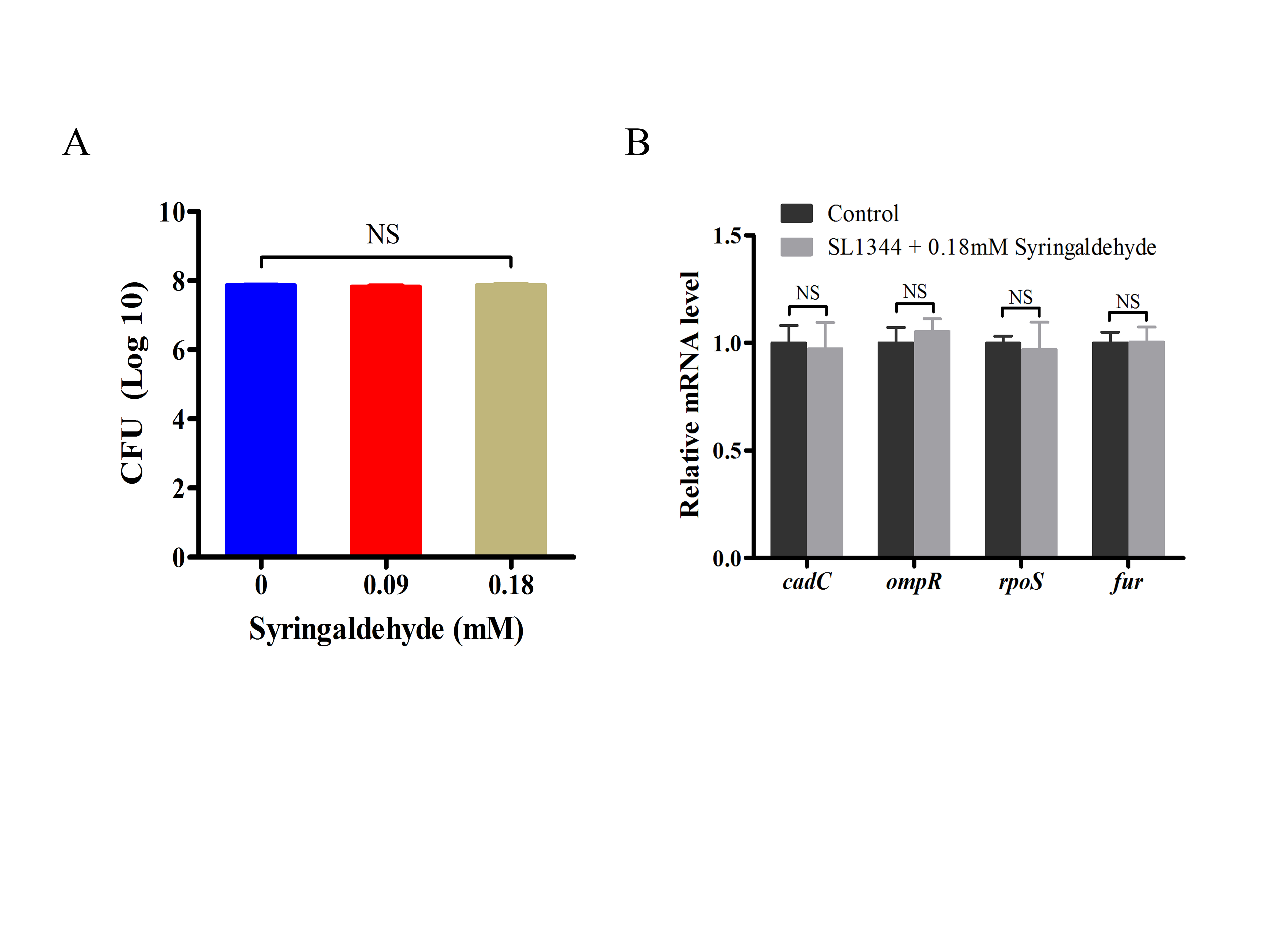

Supplement: Supplementary file 2 [file JCMM-23-4679-s002.tif]
