# Supplementary material for: Inhibition of the type III secretion system by syringaldehyde protects mice from Salmonella enterica serovar Typhimurium
Source: J Cell Mol Med. 2019 May 8;23(7):4679–88. doi: 10.1111/jcmm.14354 (PMC6584516; doi:10.1111/jcmm.14354)
Supplement: Supplementary file 3 [file JCMM-23-4679-s003.docx]

**Table S1** Primers used for the relative mRNA levels of the major acid resistance gene of *S*. typhimurium..

| **Primer** | **Sequence (5’-3’)** | **Product size (bp)** |
| --- | --- | --- |
| *fur*-Forward | *CCTGATCGACATGGGTGAAG* | 170 |
| *fur*-Reverse | *CTTTTCCGCAATCAAGGCAG* |  |
| *ompR*-Forward | *TGGTCTGTCGATTTGTCGTC* | 70 |
| *ompR*-Reverse | *CTTCGCCGTGACCATAATGA* |  |
| *rpoS*-Forward | *ATAAACTGGACCACGAACCG* | 115 |
| *rpoS*-Reverse | *GGGGTGTCTACCGAGGTAAT* |  |
| *cadC-*Forward | *CTGCTGAACACCTACTCGAC* | 115 |
| *cadC*-Reverse | *GATAGTGGCGCTGATTGACA* |  |
| *gyrB*-Forward | *TCATTTCCACTACGAAGGCG* | 111 |
| *gyrB*-Reverse | *CCGATACCGTCTTTTTCGGT* |  |

**Supplemental Figure Legends**

**Figure. S1** Effect of syringaldehyde on the adhesion and intracellular replication of HeLa cells by *S.* Typhimurium.

A. The effects of syringaldehyde on the *S*. Typhimurium adhesion of HeLa cells. Wild-type *S*. Typhimurium SL1344 treated with syringaldehyde was used to infected Hela cells.

B. The effects of syringaldehyde on the *S*. Typhimurium intracellular replication of HeLa cells. The results shown are from one representative experiment performed in triplicate. “NS” represents no statistical difference.

**Figure. S2** Effect of syringaldehyde on the tolerance of *S*. typhimurium to gastric acid.

A. The effect of syringaldehyde on the survival level of *S*. typhimurium in artificial simulated gastric fluid *in vitro*. Briefly, the overnight bacterial co-culture with or without syringaldehyde at 37 °C for 4 h. Then diluted it with 0.1% peptone water to a concentration about 10^8^ CFU/ml, and 200μl of the suspension was transferred into a prewarmed bottle containing 800μl of SGF (8.3 g/l proteosepeptone, 3.5 g/l D-glucose, 2.05 g/l NaCl, 0.6 g/l KH2PO4, 0.11 g/l CaCl2, 0.37 g/l KCl, 0.1 g/l lysozyme, and 13.3 mg/l pepsin) at 37 °C for 20 min, and the survival level of bacteria were counted by plating on LB agar plates.

B. Effect of syringaldehyde on the relative mRNA levels of the major acid resistance gene of *S*. typhimurium.

The results shown are from one representative experiment performed in triplicate. “NS” represents no statistical difference.
